# Human augmentation of ecosystems: objectives for food production and science by 2045

**DOI:** 10.1038/s41538-018-0026-4

**Published:** 2018-09-21

**Authors:** Masatoshi Funabashi

**Affiliations:** Sony Computer Science Laboratories, Inc., 3-14-13 Higashi-Gotanda, Shinagawa-ku Tokyo, 141–0022 Japan

**Keywords:** Environmental impact, Community ecology, Nutrition, Environmental biotechnology, Metabolomics

## Abstract

Current food production systems require fundamental reformation in the face of population growth, climate change, and degradation of health and the environment. Over the course of human history, every agricultural system that has emerged has featured some sort of trade-off between productivity and environmental load. These trade-offs are causing the planet to exceed the boundaries of its biogeochemical cycles and are triggering an unprecedented extinction rate of wild species, thus pushing global ecosystems to the brink of collapse. In this era, characterized as it is by human activity that can profoundly influence climate and the environment (i.e., the Anthropocene epoch), tipping points can be either negative or positive. While a negative tipping point can produce sudden, rapid, and irreversible deterioration of social and environmental systems, a positive tipping point can produce improved health and sustainable social-ecological systems. The key to promoting positive global tipping points is a thorough understanding of human activity and life history on an evolutionary scale, along with the comprehensive integration of science and technology to produce intelligent policies and practices of food production, particularly in the developing world (See Supplementary Material 1 summary for policymakers). Simply increasing the efficiency and scale of monoculture-intensive agriculture is unlikely to drive social-ecological change in a positive and sustainable direction. A new solution to the health-diet-environment trilemma must be developed to achieve a net positive impact on biodiversity through the anthropogenic augmentation of ecosystems based on the ecological foundation of genetic, metabolic, and ecosystem health. This paper discusses the fundamental requirements for sustainable food production on the molecular, physiological, and ecological scales, including evolutionary and geological insights, in an attempt to identify the global conditions needed for the primary food production to ensure we survive this century. Particular emphasis is placed on how to make extensive use of this planet’s genetic resources without irretrievably losing them.

## Introduction

The history of food production is characterized by some of humankind’s most brilliant achievements along with a questionable environmental legacy. Thanks to the development of agriculture more than 10,000 years ago, and advances in agricultural technology made possible by the industrial revolution, today’s human population has increased to 7.5 billion. These advances have also created civilizations and advanced social systems capable of influencing global ecosystems.^1^ While the green revolution may have saved the most human life ever,^2^ extensive use of synthetic fertilizers, pesticides, and high-yielding crop varieties has created global footprints that could trigger the planet’s sixth massive human extinction event.^3^ This brings into question the sustainability of primary food production in terms of material resources and both environmental and human health. Already, the biogeochemical flow of agricultural inputs such as nitrogen, phosphorus, and carbon is exceeding planetary limits, and the irreversible loss of genetic diversity could dramatically alter the integrity of the biosphere.^4^ Healthcare expenditure has become one of the heaviest economic burdens in both developed and developing countries.^5^ The overall problem is linked with conventional food production systems and the diet-environment-health trilemma, where only two of these three options can be selected.^6^ A return to a sustainable trajectory requires a drastic and fundamental reformation of conventional food systems over the entire value chain of production, distribution, and consumption.^7^

**Fig. 1:**
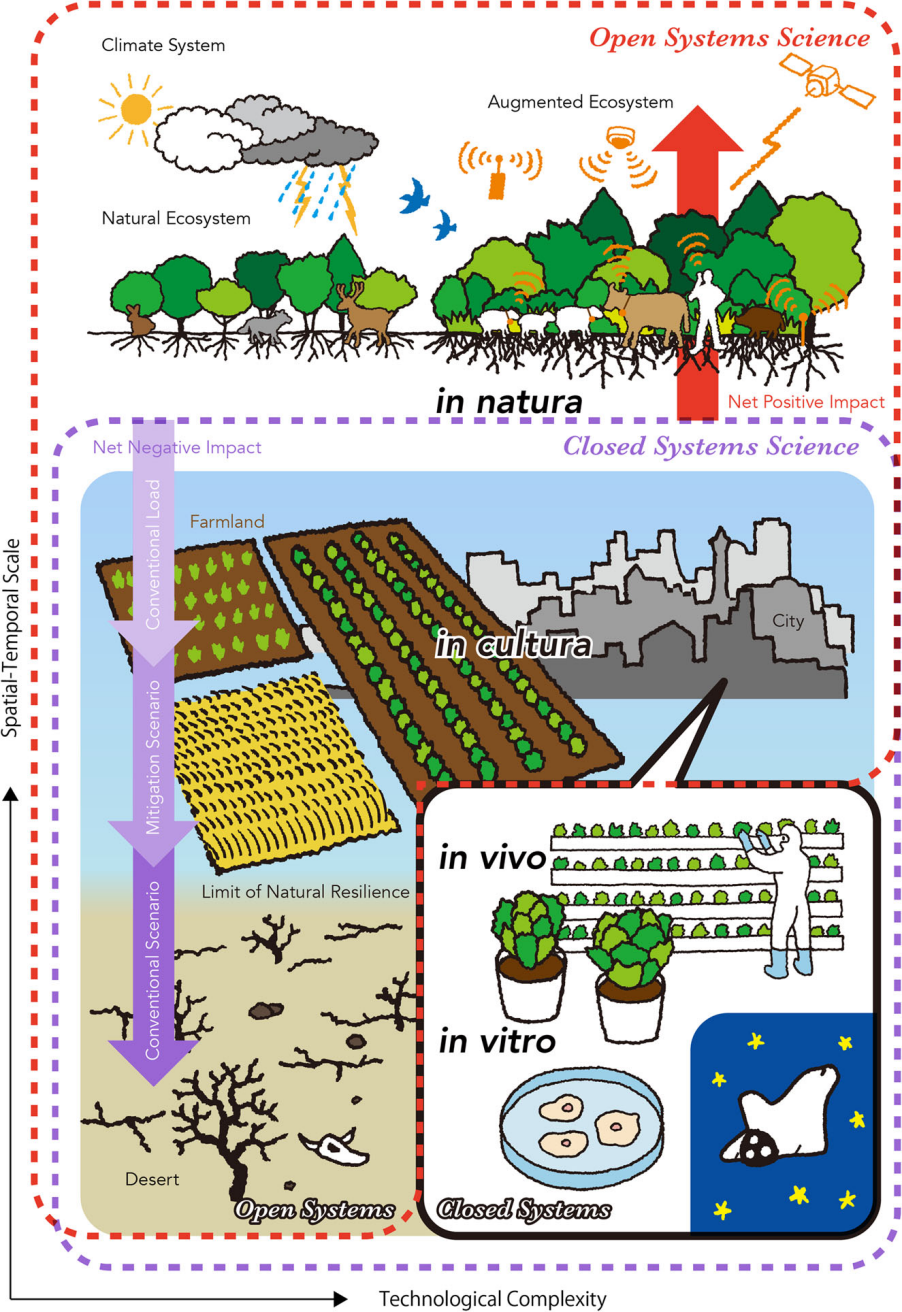
Scale of scientific and industrial domains and associated human impacts on food production. Horizontal axis represents the degree of technological complexity required for realization. Vertical axis is the spatial-temporal scale involved for the maintenance from small (bottom) to large (top) scale, in which experimental systems in science and production modes in industry can be represented as *in vitro*, *in vivo*, *in cultura*, and *in natura* conditions. As a solution for future food production, anthropogenic augmentation of ecosystems is situated at the top right, which combines enhanced agricultural biodiversity with the support of information and communication technologies (ICT), making use of various biological resources in dense and mixed polyculture situations without external material inputs. (See more explanation in Supplementary Material 2)

The spatial and temporal scales related to food production and consumption are various, ranging from the global environment to the molecular functions involved in health. Figure 1 summarizes the relationship between scientific and industrial domains and their respective effects on the environment. Most biological studies related to food products are somewhat limited because they are produced in highly controlled laboratory conditions, such as *in vitro* tissue culture or *in vivo* tests on a small number of model organisms. This is not a critique of the methodological limitations of food science but rather a reminder of the importance of carefully selecting those factors associated with food production. The ongoing feedback between field/farm production and laboratory evaluation will become more important in wider contexts.

The actual operating scale for primary food production is the open field, with interventions ranging from monoculture farming (referred to as *in cultura*) and hunting-gathering in natural ecosystems (referred to as *in natura*^8^). For example, agronomy focuses mainly on yield optimization using monoculture (i.e., *in cultura*), while ecology principally investigates the natural formation of ecosystems (*in natura*) with various degrees of disturbance. In the ecological theory of productivity optimization, the physiological optimum is defined as the optimization of a single organism (e.g., plant or animal) grown *in cultura*, while *in natura*, it is defined as the spontaneous organization of a community, where a species grows in association with others (i.e., the ecological optimum).^9^ Modern societies are mainly situated under the strong regulations of a natural environment dictated by human activities (*in cultura*), where most of the medical cohort studies are performed. The loss of natural ecosystems under human population pressure has increased consistently throughout the history of agriculture, just as *in cultura* conditions dominated over *in natura* conditions in the human habitat and agricultural landscape. Changes in global biogeochemical cycles as a result of human activity is referred to as the Anthropocene epoch.^10^

Figure 1 shows the range and types of consequences that can arise spatially and temporally (*y*-axis) as a function of the increasing complexity of technology (*x*-axis). While conventional scenarios of city and farmland development would eventually lead to a global state shift typically represented as desertification,^1^ innovative technologies for future food production would tend to focus on controlled environment solutions such as autonomous farming and plant factories, similar to *in vivo* experiments^9^ and *in vitro* cultured meat production.^11^ These emerging alternatives are based on highly controlled and confined environments compatible with cGMP laboratories and, in a most extreme case, with spacecraft on interplanetary journeys.

Building on studies using closed systems (e.g., laboratories), conventional science has been extending its reach to applications in the open environment, which features multiple external loads (area inside purple dotted line, Fig. 1). Here, there is a serious need for a better management framework, particularly with a novel formalization called open systems science (area inside red dotted line). Open systems science puts strong emphasis on relationships with surrounding systems when solving problems and makes extensive use of computational technologies to dynamically update the definition of the problem domain, its structure, and its functions so as to deliver timely and effective control within limited observation conditions.^12^ Management of *in natura* ecosystems under a given climate condition has the potential to enable an augmented state of biodiversity through the application of open systems science and the development of supportive information and communication technologies (ICT) such as satellite remote sensing, cloud computing, artificial intelligence, and internet of things (IoT)^13–16^ (top right, Fig. 1). This is qualitatively different from so-called “smart agriculture” based on the application of ICT to conventional methodologies, since the augmentation imposes the net increase of ecosystem functioning beyond natural organization.^9^ Generally, the ecological optimum is the description of an eventually established form of ecosystem based on the assumption that the evolutionary nurtured self-organization capacity is performing some kind of comprehensive optimization of ecosystem functions at the community level (i.e., naturally formed ecosystem multifunctionality^17^). Due to the nature of ecosystems that develop in harmony with the vegetation, the diversity of ecological transitions, and the existence of alternative stable states,^18,19^ ecosystem functions that occur with the composition of different species under the same environmental condition could be different and bring different levels of ecosystem services. This implies that, by the manipulation of artificial species diversity, the ecological optimum has an expandable range compared to naturally occurring ecosystems.

The Y-dimension in Fig. 1 (purple and red arrows) represents the degree of human impact with respect to negative (downward, purple) and positive (upward, red) effects on biodiversity. Effective mitigation scenarios should prevent the environmental load from exceeding natural resilience, which requires the augmentation of ecosystems towards higher *in natura* organization, in order to cancel out total loss and achieve net positive impact.^9,20^ Often, the offset argument of biodiversity and carbon neutrality mistakes the basal line as the conventional load instead of natural state: for example, the much-touted high biodiversity in farmland is not necessarily higher than the natural preservation state, which is lost anyway during the initial land conversion to farmland, which accounts for 1/6 of the annual greenhouse gas emission on the planet^21^ and is the principal driver of mass extinction (more so than the extinction pressure of climate change^22^).

Understanding the environmental load and possible mitigation and resilience measures requires a multi-scale integrative framework of the complex systems involved. Since the conventional load is estimated to impose social and ecological state shift by the middle of this century,^1,23^ the offsetting and recovery of material cycles and biodiversity with a proper evaluation regime is the baseline task. This includes foresight toward sustainable intensification in food production, including a shift of culture and food systems to a vegetarian diet with respect to forest protection;^24,25^ the amelioration of inappropriate production and distribution;^26^ the enhancement of local production in urban agriculture;^27^ the search for ecologically and nutritionally sound alternative diets such as insect food;^28^ the diverse tailoring of small-scale agriculture for resource-poor farmers;^29^ and the planned management of fishery resources based on the functional capacity of ecosystems.^30^ Moreover, the measures should incorporate the resolution of both environmental and health risks beyond mitigation so as to achieve a net positive impact and compensate for the population increase and social inequality.^6,9,20^

The integrated approach is also crucial in light of climate change. Agriculture, forestry, and other land use related to food production account for 25% of global human-caused greenhouse gas emissions.^31^ The countries most vulnerable to climate change are also more likely to foster social corruption,^32^ hunger,^33^ and biodiversity hotspots, especially in coastal areas.^34^ Primary food production in these countries is supported mainly by small-scale, family-owned farms, which occupy about 87% of the world’s agricultural land.^35^ Globally, small and medium farms produce up to 77% of major commodities and nutrients.^36^

These problems are clearly out of the reach of conventional food production optimization techniques such as precision agriculture, which is based on the business-as-usual scenario and R&D investment policy in developed countries. For example, only a 1–3 percent increase of net returns is reported through the adoption of precision agriculture by large farms in the U.S.^37^ In European countries, despite partial reduction of conventional inputs, alternative costs for implementation rise, and no profitability has been demonstrated so far in the investigated cases and scenarios.^38^ Since it is qualitatively based a priori on the negative impact on the environment, the scaling-out of monoculture solutions cannot neutralize the adverse ecological effect in the context of increasing world population and the anticipated global collapse of ecosystems.^39^ Even leading-edge agricultural technologies such as genetically modified crop production cannot ensure secure and sufficient biodiversity for essential regulation services, which has been exclusively nurtured by the natural vegetation holding an astronomical number of active genetic resources. Genetically modified organisms (GMO) in the open field rather entail a potential risk of gene diversity homogenization through crossing with wild relatives and interspecific gene transfer as a generic mechanism of genome evolution.^40^

Global synthesis of scientific knowledge has resulted in continuous alerts on the falling trajectory of the Earth system over the past 25 years.^41^ Sustainable food production, if such a category could exist, should incorporate the overall trade-off and potential synergy of multi-scale interactions among *in vitro-in vivo-in cultura-in natura* phenomena toward the end goal of a biological diversity that is balanced with, or superior to, human activities across the whole value chain of research, policy making, production, distribution, consumption, recycling, and health effect. Designing societies that sustain an anthropogenic biosphere with nonhuman natures is crucial in the present era of Anthropocene.^42^ Altogether with the integration of agronomy and ecology, food science is poised to play a central role in the integrity of food production by substantially incorporating ecological effects and benefits derived in the long-term as an integrated life science that advocates health and abundance for both humanity and nature.

## Problems of elementalism and true causality

Current international initiatives in food science tend to focus on component analysis and effects on public health (e.g., ref. ^43^). This bottom-up extrapolation from laboratory to human body and field is common in agronomy and medicine as well, forming the basic methodology of elementalism in science. However, the quantitative expansion of current approaches does not necessarily guarantee a sure path to problem resolution. Oddly enough, as we accumulate more and more partial evidence, an increasing amount of hidden parameters are disregarded. These might comprise a myriad of contradicting microscopic evidence disconnected from the appropriate integration or could result in a global consensus that remains unaware of any influence of an unseen bias. Scientists who “can’t see the forest for the trees” may become more prevalent in studies of living complex systems. In this analogy, trees are individual omics studies and the forest is the integrated principle of human and ecosystem health. A typical example is the worsening R&D efficiency in the pharmaceutical industry.^44^

To put it bluntly, high-throughput discovery-oriented research using only statistical tests has methodological pitfalls. Post-study probability that the claimed result is actually true could be extremely low, even in established areas with empirical evidence supported by expert opinions.^45^ Indeed, the significance level *α* = 0.05 of *p*-value only defines the type I error to detect a false positive result, which means that any null hypothesis could be rejected over the course of 20 experiments. This would result in at most 95% false positive results in published articles, which is not very far from actual irreproducibility of clinical drug trials (take for example the area of cancer research, where 89% of landmark publications could not be replicated^46^).

On the other hand, a truly significant effect might not necessarily be detected if the statistical power is low. Not only the statistical results but also a true relationship that bears the principle of Occam’s razor (i.e., the simplest theory that satisfies the necessary and sufficient condition for the explanation of a phenomenon) should be contextualized within the complexity of biological systems. As a general property of biochemical pathways and gene interactions, physiological responses emerge as a combined effect of various elements: bioactive compounds usually exhibit a non-linear dose effect that acts positively in the middle range but becomes lethal in both deficient and excessive quantities; interactions of multiple compounds form a kind of fuzzy logic that accepts the ambiguity of the biological reactions depending on each dose; the activation of a downregulation pathway could reverse the physiological consequence in downstream receptors; and high-order correlations on more than two variables are hugely unexploited in biological studies.^47^ As a system-level property, these interactions could be a part of the necessary conditions for the survival of an organism, but they are not necessarily detectable in component-wise study (i.e., false rejection of elements that form part of the true necessary condition). Moreover, the robustness of biological systems means they also accept marginal properties that are not necessarily correlated with the survival rate but still show significant difference in adaptive characteristics (i.e., false acceptance of unnecessary conditions).

A key underlying task here is to distinguish true causality from observed correlations that might be just an accidental association or pseudo-correlation. Significant correlation may also act as a noise and hinder the discovery of hidden causality. Statistical analyses usually do not distinguish between pseudo-correlation and true causality, which could deliver compromising results. As for micronutrients important for long-term health protection, antioxidants are a typical example of such complexity of combined effect: *in vitro* and *in vivo* evidence of flavonoid shows significant antioxidative effects at the cellular level^48^ and is contained in abundance in natural products in the food system where life expectation is long,^49^ even though supplements of other antioxidants such as beta carotene, vitamin A, and vitamin E have actually increased the mortality of healthy and stable-phase patients in large clinical trials.^50^ Such discordance arises from taking blindly each food component and physio-chemical property as the necessary condition of systemic health. What if the significance of pseudo-correlation and true causality depended on different scales? Food components and metabolic markers are certainly correlated, though latent causal variables may exist outside of the measurement, which could be the common source of observed pseudo-correlation. Statistical correlation between events A and B does not necessarily imply causality, and it is an open question whether some latent variable C could exist that simultaneously affects both A and B as a true causality. A typical example is the culture condition of produce that affects plant metabolism and other food variables associated with the health effect of consumers.^9^ A nutritive element highly correlated with health state could be just a co-occurring marker of other true causal factors outside the scope of measurement, or just a part of the causal factors that should interact simultaneously to exert observed effect.^8^ This is what necessitates the endless validation processes in food science. The challenge of determining causal elements dramatically increases the difficulty when evaluating the health effect of food items as a whole, outside the scope of component-wise intake testing.

**Fig. 2:**
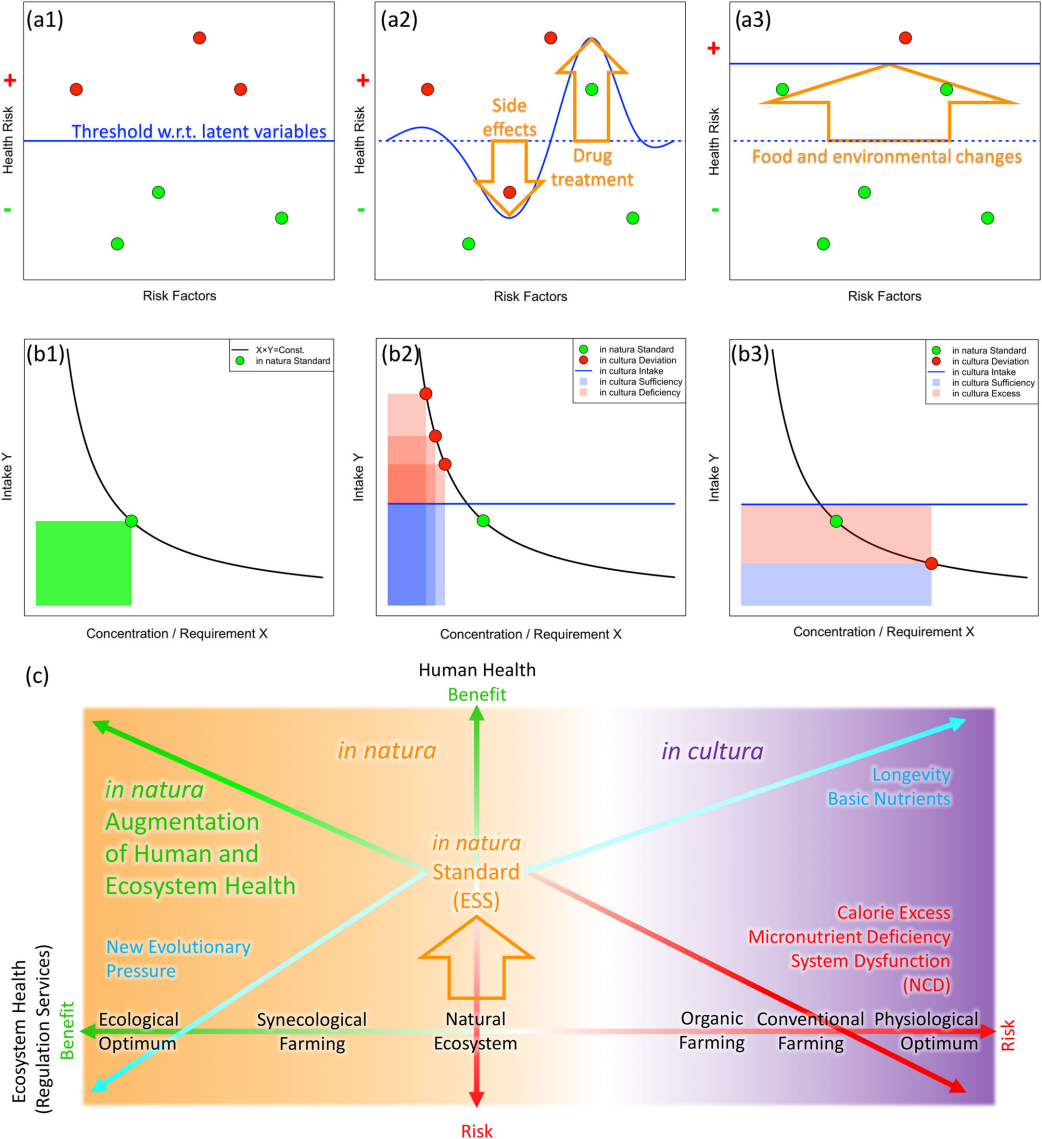
**a1**–**a3**: “Hidden reef model” that integrates observable (red and green circles) and latent (blue line) variables in biological study. **b1**–**b3**: Balance model of food variables with respect to evolutionary stable state (ESS, set as the green circles). *x*-axis is the concentration (content per unit weight) of food variable divided by physiological effects or environmental requirement for production. *y*-axis is actual amount of the food intake. **c** Relationship between human and ecosystem health and farming methods. Intensification toward the ecological optimum beyond the natural state, such as synecological farming (ref. ^8^), creates a new integrative approach that has the potential to address both human and ecological health as positively interacting solutions (upper left green arrow). (See more explanation in Supplementary Material 3)

Such complexities underlying the evidence construction create the burden of “devil’s proof” in obtaining an integrated view beyond experimental conditions and data limitations. One cannot test a hypothesis that distinguishes true causal factors without a sufficiently comprehensive setting that extensively involves potentially related variables. Separated efforts in different disciplines methodologically omit the possibility of discovering a unified framework where the variables in different fields are coupled and mutually affect each other in real situations. In order to ease this burden and establish a multi-scale integrative model, various terminologies in the food and medical sciences such as “risk factors” and “beneficial components” should be integrated with explicit representation of latent variables in the background, represented in Fig. 2 (a1–3) as the “hidden reef model”.

In the hidden reef model, from a systems biology premise, complex living organisms with various feedback possess memory structures such as internal metabolic states that provide the context of biological response to a given treatment. Such internal state could be influenced by genetic variability, environmental factors, social status, and other random mechanisms that are usually difficult to measure and standardize.^51,52^ In Fig. 2 (a1), the observables related to an organism’s health state can analogically be called a “reef” that is partially submerged below the “sea surface” (blue line). The level of the sea surface represents the health effect threshold that integrates the net effect of latent variables outside the scope of measurement, such as internal metabolic state and environmental conditions. The sea surface refers to the underlying measurement conditions, which are different among *in vitro,* *in vivo, in cultura*, and *in natura*. A part of the reef may or may not be above the sea surface, representing the health risk (red circles) or benefit (green circles), respectively. (a2) shows the case of a drug treatment that partially modifies the sea surface and reduces the risk of a specific target while producing a side effect in other factors. This can be analogically described as the reef in a rough sea situation, with the wave representing a drug surge. It can also represent individual variation of susceptibility to disease under the same value of observables. (a3) represents ideal treatment that raises the sea level by changing the environment, or moving the reef downward by modifying individual traits, so that most of the reef can sink below the risk threshold. Evolutionary stable state (ESS), which forms the robustness of genetic and metabolic networks, can be considered to converge toward such a “hidden reef” state under *in natura* dietary and environmental conditions.^53^ Examples of the observable (reef) and latent (sea surface) variables are given in Supplementary Material 5.

This formalization can situate classical problems in genetic pathology such as missing heritability^54^ in relation to environmental factors such as diet pattern. It can also express phenotypic variability in experimental animals as the background variation under the same genetic profile.^51^ Usually, the sea surface is implicitly set as a flat threshold in each individual study. However, mapping the relationship between different measurements requires reference to latent variables, where the expression of a variable sea surface is required as an interface between different experimental systems. The condition for the sound interpretation of the emerging omics studies toward the amelioration of long-term health and well-being is the integration of multi-scale latent variables, from molecules to environments, that are cut off in each study but become essential in real situations.

While health problems of simple causality such as nutrition deficiency and excess are easy to address, recently emerging non-communicable diseases typically involve multi-scale variables from genetics to environment (such as diet and lifestyle) that make up the dysfunction of the whole system rather than the defect of a specific component.^5^ These variables form multiple feedback loops between food, endocrine and nervous systems, gut microbiota, and surrounding ecosystems, and we currently only have a partial understanding of these metabolic pathways (e.g., ref. ^55^).

Resolving system dysfunction in living complex organisms ideally requires the simultaneous and identically precise measurement of all variables, but essential dynamics can be secured by appropriately choosing the scales of modeling. In other words, scientific methodologies are strictly based on reductionism, but the elements to which we reduce the phenomena can be chosen in multiple scales of the system’s hierarchy. By appropriately choosing the descriptive variables from different hierarchies of the interacting systems, we should be able to integrate the important effects of latent variables that are difficult to cover with single omics study. The challenges and development of reductionism in the face of complex system problems can be summarized into three steps: elementary reductionism, systems theory in a confined environment, and live management of open-ended complex systems (Supplementary Material 6).

## Interface for integrated life science *in natura*

Let us consider the evolutionary scale *in natura* that formed our own genetic and metabolic profile.^53^ Natural selection could theoretically lead to the realization of evolutionary stable state (ESS) as a genetic composition supporting long-term fitness under a moderately fluctuating environment.^56^ Such a condition for modern *homo sapiens* can be traced anatomically to roughly 300,000 years ago,^57^ preceded by the adaptation of basic metabolic systems to the hunter-gatherer lifestyle over several million years, until the recent shift to agrarian life. Lifestyle and dietary modification supported by agriculture and consequent environmental change in Anthropocene have generated the complexity of food production on which various scientific disciplines are currently divided (Fig. 1).

In order to integrate the relevant disciplines to resolve the diet-health-environment trilemma, sciences that support sustainable food production should incorporate the original cause as an interface: that is, the abrupt change of the food culture condition from *in natura* to *in cultura* with agriculture. We should then consider how such a macroscopic condition has led to the diversification of microscopic symptoms as observed in public health and environmental problems, where each discipline struggles without necessarily having reference to others.

Figure 2 (b1–b3) shows how the *in natura-in cultura* distinction could serve as an interface to explain the diet-health-environment trilemma, specifically, as a balance model of food variables with respect to evolutionary stable state (ESS) incorporating the hidden reef model in Fig. 2 (a1–a3). In (b1–b3), the *x*-axis represents the quantity of food variables per unit of food item divided by the physiological or environmental requirement (nutritive contents, environmental effects of the production, etc.). In the case of nutrients, the value of concentration on the *x*-axis corresponds to the nutritive content per unit of food weight, which coincides with the multiplication of nutrient density by energy density commonly used in dietary guidelines.^58^ The *y*-axis shows the quantity of actual food intake, such as food weight. In (b1), all food variables and intake quantity are normalized to the green circle representing the *in natura* ESS as the standard point on the nutrition need curve *x* × *y* = Constant, where the constant is the nutrition profile in natural state. Conversion to an *in cultura* environment represented in (b2, b3) will diversify the parameters of each food variable (red circles), which affects the net physiological requirement of an individual organism (upper shift of blue line above the green circle). The required intake level (*y*-value) of the red circles is set to meet the demand in terms of the green circle, while that of the blue line represents the consequent response of an individual organism.

As a typical example of malnutrition, micronutrient deficiency (left shifts of red circles in b2) in high-calorie food (right shift of red circle in b3) that leads to overeating (upper shift of blue line) is depicted. Red-shaded areas in (b2) and (b3) represent the amounts of micronutrient deficiency and excess calorie intake, respectively. This upper shift of the blue line is adaptive in two senses: one, animals tend to eat more food than necessary when it is abundant, and two, low micronutrient content necessitates increased intake to meet nutritional needs. The evolution of human feeding behavior in the ecological context also supports the diet choice for energy-dense and palatable food.^59^ As an example of a food composition study associated with health risk, the values of the *x*- and *y*-axis of the red circles correspond to the “reef” while the degree of deviation of the blue line from *in natura* ESS (green circle) is another expression of the “sea surface”. The key idea here is that the same *x*- and *y*-values of a nutrient can change their meaning for individual health between the appropriate dose of intake and deficiency/excess, according to the position of the blue line.

In the case of representing the environmental load of farming with red circles in (b2, b3), *in cultura* sufficiency, deficiency, and excess should read necessary, reduced, and excess load, respectively. The left shift (b2) can produce more food with less environmental impact than hunting-gathering, while the right shift (b3) imposes a higher rate of ecosystem degradation per unit of food. The blue line in this case corresponds to increased agricultural yield. Examples of the food variables and *in cultura* responses are given in Supplementary Material 7.

Setting the intake balance of ESS as the ecological optimum *in natura*, the *in cultura* deviation can be expressed as both deficiency and excess of food variables, leading to a total increase of health risk and overload to the environment. The necessary and sufficient conditions for both human and environmental health may reside in the reintroduction of the *in natura* state to primary food production, thus sustaining the ESS of our metabolism in the augmented diversity of ecosystems.^9^ Such an approach to primary food production is compatible with the net positive impact approach to biodiversity,^20^ which could conceivably be introduced to the world’s smallholders for a bottom-up resolution of the health-diet-environment trilemma.^60,61^

The relationship between human and ecosystem health is schematized in Fig. 2(c), providing reference to the modes of agricultural production and consequent health benefits and risks. Historically, the development of farming systems toward a physiological optimum of monoculture methods achieved higher longevity and amelioration of basic nutrition status (upper right cyan arrow) while simultaneously producing an imbalance in micronutrient profiles and calories in foods as the risk factors of non-communicable diseases and the serious loss of regulation services in farming and surrounding ecosystems (lower right red arrow). The ecological optimum solution, which increases the functioning of the ecosystem through human introduction of biodiversity in the natural development of plant communities for food production, indicates the potential to synergistically contribute to human and ecosystem health (upper left green arrow). It generates diverse functional complementarity and synergy in multiple-scale adaptation processes ranging over the physiology of each organism and the ecology of self-organized communities.^9^ Associated risks of the augmentation scenario may appear as new evolutionary pressure regarding food and ecosystem changes (lower left cyan arrow): for example, fertility decline and aging population brought on by improved values of the human development index (HDI);^62^ the emergence of trans-generational incompatibility with new food habits;^63^ conflicts between human society and protected wildlife in highly developed ecosystems, and the emergence of newly evolving pathogens.

## Preventing and reversing agriculture-induced regime shifts with *in natura* augmentation of ecosystems

What are the likely outcomes in Anthropocene if we choose to reform food production? Through the propagation of agriculture, the dominant forces that influence the functioning of the Earth system seem to have shifted from astronomical and geophysical forces to an anthropogenic driver, which entails the risk of leaving the glacial-interglacial limit cycle of the late Quaternary.^64^ Regaining and strengthening the *in natura* dynamics in primary food production will be a necessary methodology to countermeasure the anticipated regime shifts such as the collapse of global biodiversity^1^ and the deterioration of social services and the health state.^23^

Through empirical practices, evidence has shown that human activities can be exploited to enhance the biodiversity under an *in natura* organization known as ecological optimum, more so than the natural preservation state beyond the environmental trade-off.^13,60,61,65,66^ The *in natura* augmentation of ecosystems through primary food production has resulted in recent bold examples that incorporate the qualitative shift of environmental impact from negative to positive, as discussed below.

High biodiversity farmland and some elements of natural farming are known to provoke higher biodiversity in the surrounding landscape than the preservation state through positive disturbance and the introduction of new plant species.^9^ As an extreme conception, the synecoculture project (synecological farming in Fig. 2(c)) has been gathering evidence for the *in natura* augmentation of ecosystems by promoting species diversity in market gardening fields and generating high-density mixed communities with ecological optimum.^65^ Experiments in Japan have shown that as little as 3000 m^2^ can harbor crop species diversity comparable to that of the regional scale in traditional agriculture with renowned conservation value.^13^ Biodiversity records including surrounding ecosystems showed augmented conservation value including the observation of IUCN red list species, higher organization of soil microorganism diversity and associated buffering function, greater total yield, and enhanced regulation services including pollination.^61^ This means that the complete substitution of tillage, fertilizer, and chemicals with augmented ecosystem functions/services can excel in productivity as a comprehensive output of highly diverse products without losing but rather increasing soil fertility, even under continuous harvest pressure. In sub-Saharan Africa, an experiment in Burkina Faso dramatically recovered the local regime shift and reestablished the vegetation to a mature stage of primary succession in terms of species composition.^60^ The productivity was 40- to 150-fold higher than the national average of market gardening production, and total cost-effectiveness rose tenfold.^67^ These can be considered as key evidence that extend the conceivable range of sustainable development in dryland agriculture, as unprecedented high compatibility between biodiversity promotion and activation of local economy can be achieved. Synecoculture has huge potential to increase crop diversity and yield, which would minimize land clearing and eventually protect the habitats of threatened large mammals.^68^

The overall results indicate that anthropogenic forcing can be exploited to enhance the diversity and ecosystem functions of plant communities under ecological optimum growth, which can be represented as the *in natura* augmentation of ecosystems. When the biodiversity is stated to be higher than the natural preservation state, it has the following implications, according to the three divisions of species diversity:^65^

1. α-diversity, which describes the species diversity of an ecosystem at a specific ecological succession stage, can be augmented by human introduction of various crop species and consequent induction of naturally occurring species.
2. β-diversity, which describes the species diversity that corresponds to the difference between two ecosystems with different ecological succession stages, can be diversified by the separate management of multiple ecosystems with different α-diversities.
3. γ-diversity, which describes the diversity of species at all present ecosystems with various succession stages, can be enhanced through the combination of multiple augmentation and preservation states, such as through interactions between farming and the surrounding ecosystems.

The three levels of species diversity can be managed to reach a higher targeted state faster than natural preservation by means of active introduction of new species and positive disturbance through human intervention. This process may include not only the contribution of introduced non-invasive species but also positive aspects of new predominating species often dismissed under the negative label of invasive species.^69^ Since the diversity of the succession stage generally decreases through convergence to a climax phase in natural development, human management can contribute to higher biodiversity through the coexistence of various types of ecosystems. The recovery process of ecosystems is particularly compatible with human-assisted biodiversity promotion.^60,70,71^ Indeed, lightly to intensively used secondary vegetation is globally reported to excel in both species richness and abundance compared to primary vegetation.^72^ While 90% of the world’s food calories are estimated to derive from only 30 crops, historically used edible plant species number more than 30,000 (ref. ^73^), suggesting a vast untapped repertory of plant genetic resources for the augmentation of agroecosystems.

**Fig. 3:**
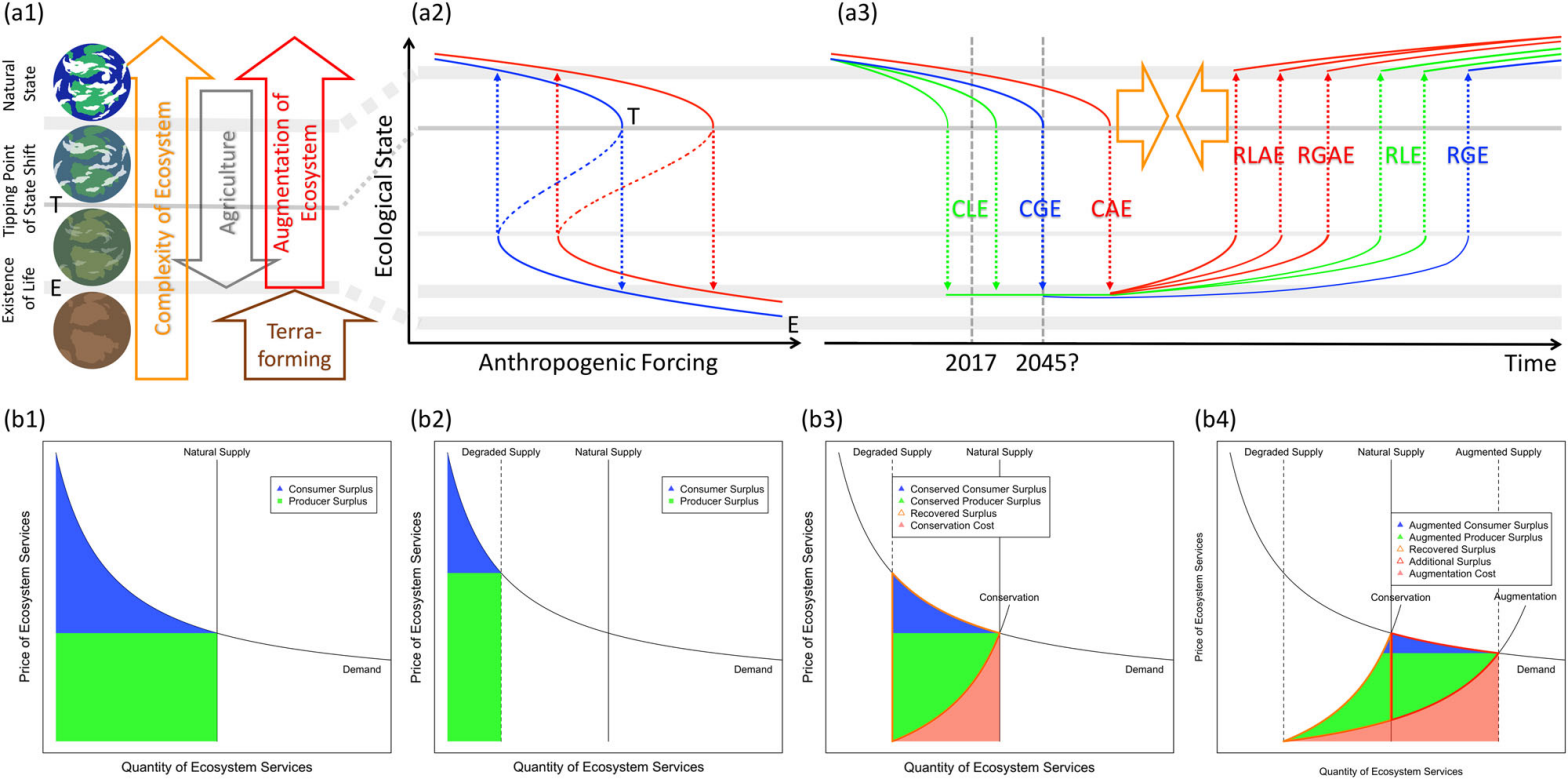
Possible scenario of prevention and reversal of ecological state shift (**a1**–**a3**) and expected outcome on ecosystem services (ES) (**b1**–**b4**). (See more explanation in Supplementary Material 4.)

One potential outcome of the Anthropocene trajectory, if we succeed in achieving such augmentation of ecosystems through the majority of primary food production, especially on the part of smallholders in the developing world,^35,36^ is that it could be a major driving force to sustain our social-ecological systems. Figure 3 (a1–a3) shows a possible mechanism and scenario for the prevention and reversal of the ecological regime shift. (a1) depicts an Earth system that has evolved toward higher complexity of ecosystems to harbor diverse forms of life (orange arrow), though agricultural forcing of *in cultura* has historically degraded biodiversity and impaired the natural material cycle (gray arrow), approaching the catastrophe threshold of a global regime shift (line T, the limit of natural resilience in Fig. 1). Augmentation of an ecosystem *in natura* could be a driving force that reconstructs ecological complexity (red arrow), which follows the same direction as terraforming, where a planetary environment (brown arrow) is transformed into a habitable condition^74^ (line E).

In (a2), the blue curve describes the general hysteresis of an ecosystem with respect to *x*-axis: human impacts such as agricultural land use change, environmental pollution, and other habitat destruction, and *y*-axis: ecological state such as biodiversity per unit surface. It is a typical phase diagram of the ecological state shift based on ref. ^1^. The red curve represents a speculation of the augmented ecosystem where some percentage of human activities are proportionally invested to promote the ecological state, reflected as a right shift of the response curve. Dotted arrows represent regime shifts between alternative stable states with biodiversity loss (downward) and its recovery (upward), which follow irreversible phase transition processes.

(a3) shows the estimated dynamics of the prevention and reversal of the ecological state shift. *x*-axis represents time in yearly scale, with the speculation of global state shift around 2045.^1^ *y*-axis is identical to (a2). Solid lines show the dynamics through time following the phase diagram (a2). *y*-value represents the biodiversity of local ecosystems (green solid lines), global ecosystems (blue solid lines), and locally or globally augmented ecosystems (red solid lines). Dotted arrows indicate the phase transitions between the two levels of alternative stable states: Collapse of Local Ecosystems (CLE, green downward arrows); Collapse of Global Ecosystem (CGE, blue downward arrow); Local or Global Collapse of Augmented Ecosystems (CAE, red downward arrow); Recovery of Local Ecosystems (RLE, green upward arrows); Recovery of Global Ecosystem (RGE, blue upward arrow); Recovery of Locally Augmented Ecosystems (RLAE, red upward arrows); and Recovery of Globally Augmented Ecosystem (RGAE, red upward arrow).

As the figures imply, augmented ecosystems (red lines) tend to mitigate biodiversity loss and lessen the gap of regime shift (orange arrows), possibly preventing it if significant augmentation took place in the majority of primary food production.

Figure 3 (b1–b4) shows the expected yield of ecosystem services with pricing mechanisms under different scenarios of development, conservation, and augmentation. All figures represent the supply-demand curve of ecosystem services according to ref. ^75^ with *x*-axis: Quantity and *y*-axis: Price.

- (b1) is the case of natural supply without human-induced degradation. Unlike conventional representation in economics, the supply curve of ecosystem services that takes constant quantity regardless of human demand is expressed as a vertical threshold line. The blue and green areas represent the amount of consumer surplus and producer surplus, respectively.
- (b2) shows the case of degraded supply under anthropogenic forcing. The supply curve shifts to the left side, causing a shrink of consumer and producer surplus.
- (b3) is a typical conservation scenario where humans pay a cost for the recovery of natural ecosystems to the level of natural supply. Conserved consumer and producer surplus (blue and green areas) in return to conservation cost (red-shaded area) can be expected as the recovered surplus (orange-bordered area).

The conventional concept of biodiversity usually puts the natural preservation state as the highest standard and discusses human-induced degradation. In contrast, the augmentation of ecosystems can create a new field of operational species diversity, including the actual establishment of augmented ecosystems and virtual diversity of knowledge.^66^ The capacity of exploitable utility also increases accordingly, as modeled in (b4): the augmentation of ecosystems beyond conservation takes a premise of realizing augmented supply of ecosystem services more than natural preservation state. In a successful case, newly recovered surplus beyond the conservation scenario (orange-bordered area) and further additional surplus (red-bordered area) can be expected as the augmented consumer and producer surplus (blue and green areas), with more cost-efficient investment (red-shaded area).

This classification of the cost and surplus of ecosystem services provides a basic market mechanism for policy making in a green economy and makes it possible to subsidize activities with a clear distinction between ecological augmentation and conventional conservation efforts. Augmentation beyond conservation can provide a more leveraging effect on ecosystem services that can be assessable by the realized ecological state and the modality of management. Current major international initiatives aimed at mainstreaming biodiversity in foods and production ecosystems should incorporate such a framework with the prioritization of investment according to the cost benefit ratio of ecosystem services, especially with the distinction of augmentation beyond conservation. Potentially applicable projects include the promotion of diversity of food, diets, and agricultural ecosystems to improve nutrition through local adaptation;^76^ policy making and action planning for the development of nutrition-sensitive agriculture;^77^ integrated market mechanisms of carbon offset and biodiversity promotion in sustainable forest use;^78^ trans-species health initiatives with united human and veterinary medicine;^79^ the Nagoya Protocol on Access and Benefit-Sharing of genetic resources and its clearing-house mechanism;^66,80^ no net loss and net positive impact approaches for biodiversity in commercial agriculture and forestry sectors;^20^ and a government-led integrative approach to agrobiodiversity in developing megadiverse countries such as *in situ*/on-farm conservation of plant genetic resources;^81^ facilitation of the sustainable use of underutilized and neglected edible species;^82^ development of perennial varieties of major crops for increased carbon fixation and low-input cultivation in marginal land;^83,84^ and adaptation of yield and ecosystem services in variable environments by increasing plant diversity.^85^ Examples of criteria for the transformation of current initiatives to the augmentation scenario are given in Supplementary Material 8.

Primary food production other than farming also shows potential to help achieve the augmentation of field ecosystems, and should be coordinated across industries. Traditional pastoralism has created some of the most biologically diverse savannah ecosystems in marginal environments, and they require new adaptation mechanisms to cope with climate change.^86^ Recently emerging management-intensive grazing practices have been shown to rapidly sequester soil carbon to the level of native forest.^87^ Extensive aquaculture systems have the potential to collaterally enrich species diversity and abundance in water ecosystems^88^ that could consequently harbor wetland habitats for avian species.^89^ Expansion of ecologically sound alternative diets such as edible insects could positively stimulate biodiversity through sustainable use of forest environments.^28^

The degree of augmentation of ecosystems *in natura*, in exactly the opposite extreme of ecological complexity compared to the past agricultural history *in cultura*, would divide the fate of the Earth system and associated margin of natural capital, including human and environmental health.

To promote these actions with scientific evidence, trans-disciplinary approaches will become essential. Multi-scale ecological and nutrient big data^90,91^ need to incorporate more precise dynamics of ecosystem augmentation to overcome the limitations of the conventional scenario in future provision. This should go beyond the nutrition-wise risk assessment and into system-level properties that could address system-dysfunction problems in a unified body of humans and ecosystems. The merge of individually tailored precision medicine, nutrition, and public health with longitudinal multi-omics^92^ should integrate latent environmental variables (Fig. 2 (a1–a3)) and fit into the context of the expected benefits from augmented ecosystem services (Fig. 3 (b4)).

As discussed above, bridging the gap between food component diversity and ecological diversity is a technical task of immediate importance in science policy. Sustainability requires both technical and social support working in tandem to build resilience in coupled social-ecological systems. At a more fundamental level, the establishment of a sane local and collaborative economy that narrows economic disparities through equitable production and distribution modes is essential as a basic social foundation.^7^ It should also reduce waste, consumption, greenhouse gas emissions, and infrastructure expansion to a scale that could contain the impacts of human civilization to within planetary boundaries. This will require the reformation of economic activities with substantial shifts to the sharing of various goods and services in order to save and recycle non-renewable resources, replace energy-consuming lifestyles with activities that positively contribute to the environment, and provide the majority of basic commodities from augmented ecosystem services. The development of legal systems compatible with the new regulatory frameworks is needed in policy making, and for a prompt and effective implementation, extensive modalities of information and communication technologies (ICT) realizable in the era of the Fourth Industrial Revolution should be employed, in close coordination with global sustainable development goals.^15,66,93^ Technological and social intermediaries should be provided in an open-source and participatory structure to maximize accessibility and collaborative synergies among multiple stakeholders who are increasingly working as decentralized autonomous organizations (DAO). Governmental initiatives toward smart society, such as Japan’s Society 5.0, need to set the augmented ecosystems as the baseline of future natural capitals and exploit the full potential of the entire biosphere through the burgeoning capacity of cyberspace (Fig. 1, top right).

Closing the social gap and reducing the difference of environment loads between developed and developing countries would lead to a new phase of demographic transition. Specifically, the amelioration of food quality and biodiversity recovery in the developing world will help to increase the human development index (HDI), which is generally linked with the decrease of fertility. At the same time, progress in advanced HDI countries is known to rebound positively to the fertility rate.^62^ This will result in a narrower fertility gap between developed and developing worlds, leading to a more equitable distribution of human population among age groups and geographical regions with a higher and narrower range of HDI. Among factors that define HDI, health measures such as life expectancy could be substantially improved by reforming primary food production and the associated natural environment. The outcome of this would be extremely positive for Anthropocene transformed into a symbiotic Earth, where appropriately scaled human civilization and augmented ecosystems play mutually beneficial roles for the prosperity of the other.

## Conclusion

Food production requires fundamental reformation to cope with the ongoing degradation of the planet’s ecological state and associated health risks. Such restructuring cannot be done by simply promoting the efficiency of agricultural trade-off, i.e., sacrificing biodiversity for productivity, nor by combining the elementary knowledge of *in vitro/in vivo* and cohort studies under the bias of the monoculture (*in cultura*) production dominant in conventional food systems. Rather, it needs to incorporate the *in natura* dynamics that have been the global life support throughout the evolution of ecosystems and that have carved out the genetic and metabolic plan of humans and other living organisms. This multi-scale perspective should integrate and renew relevant disciplines such as molecular and systems biology, food and medical sciences, agronomy, ecology, and Earth science with the interface of ICT and computational science under the context of *in natura* life science. In order to establish compatibility between the sustainable food industries and a higher standard of public health, emergent longitudinal multi-omics studies on human health should further incorporate and distinguish macroscopic ecological variables ranging over the destruction, preservation, mitigation, conservation, and augmentation of biodiversity. Such integration requires a criterial model that contextualizes and interrelates experimental studies on different scales from individual physiology to community ecology.^9^

Given the limited time frame before the anticipated tipping point of social-ecological systems,^1,23,64^ primary food production *in situ* should not wait for these changes to occur: instead, we must proactively proceed with the augmentation of ecosystems in each location, especially in small and middle scale farmland, to maximize their economic and ecological interests in a long-term perspective taking planetary limits of material resources into account.

To promote these actions, technological investment, subsidies, and policy making should substantially shift the support to empower low-input, biodiversity-mainstreaming smallholders to generate bottom-up synergy among the majority of stakeholders. A major part of food systems that could provide fundamental life support for the estimated population of 9.1 billion in 2050 need to be realized as the *in natura* augmented ecosystems established by local smallholders. This should be associated with technological leveraging such as ICT support for the resource-efficient management of megadiversity in globally decentralized systems of production and distribution.^13–15,61^

Global sustainable development goals call for an industrial and scientific revolution, setting food production as one of the main pillars of reform without precedent,^60,93,94^ where the augmentation of human capacity and global ecological state it creates will play a decisive role in the future trajectory of the Earth system.

## Electronic supplementary material

Supplementary materials

## Electronic supplementary material

**Supplementary information** accompanies the paper on the *npj Science of Food* website (10.1038/s41538-018-0026-4).
