# Supplementary material for: Human augmentation of ecosystems: objectives for food production and science by 2045
Source: NPJ Sci Food. 2018 Sep 21;2:16. doi: 10.1038/s41538-018-0026-4 (PMC6550257; doi:10.1038/s41538-018-0026-4)
Supplement: Supplementary file 1 — Supplementary materials [file 41538_2018_26_MOESM1_ESM.docx]

**Supplementary materials**

**Supplementary material 1**

**Summary for policymakers**

This article explains why an eventual synthesis of professional silos such as agronomy, ecology, food, and medical sciences alone is not sufficient to overcome the complexity of the diet-health-environment trilemma and emphasizes the importance of driving fundamental participatory change in food production with deliberate enhancement of biodiversity and ecosystem functions to prevent the anticipated ecological regime shift and associated social collapse.

The standard viewpoint of ecologists that "better prediction leads to better decisions" is not proactive enough to prevent the global state shift because it disregards the massive developmental pressure of agriculture based on the fundamental trade-off between biodiversity and productivity.

Current rates of species extinction along with demographic pressure far outpace the innovations of monoculture-based production, such as the amelioration of yield gaps and its scaling-out strategies, which promotes technically precise resource management but is fundamentally inconsistent with biodiversity protection.

Furthermore, in order to properly situate a variety of evidence in a unified perspective of human and environmental health, recently emerging multi-scale omics studies in food and medical sciences need to incorporate the conditions that affect the metabolite of products and the associated loss and formation of biodiversity—typically, the culture conditions of crops and the various ecological factors that support food production.

In the past, traditional agricultural societies were able to achieve major relief from food shortage by producing a wide variety of comestible plants and livestock through breed improvement. However, this came at the cost of enormous burdens related to input-intensive production—resource depletion, the destruction of ecosystems and erosion of topsoil, and the consequent deviation of products’ metabolite from the evolutionary nurtured state—all of which imposed metabolic aberration on humans.

The integrative countermeasure to these problems, known in the field as the anthropogenic augmentation of ecosystems through primary food production, aims to utilize the positive legacies of agrobiodiversity and assistive technologies in an integrative cycle of human and ecosystem health. It coincides with important international incentives of environmentally responsible development, such as the mainstreaming of biodiversity in food production, *in situ*conservation and the exploration of untapped plant genetic resources, and further extends its reach towards dynamic adaptive strategies for the establishment of highly functioning, useful, and valuable ecosystems. The augmentation scenario can be distinguished from conventional scenarios of reducing environmental load (preservation, conservation, mitigation etc.) in terms of the enhanced level of biodiversity and ecosystem services compared with an uncontrolled natural ecosystem, and high compatibility with local economic activities based on sustainable utilization of natural resources.

To establish effective policy-science interfaces, links to exact scientific terms and references are provided to facilitate the crucial coordination of cross-sectional task forces that should work to oversee the efforts of existing expert committees. Under such transdisciplinary and integrative measures, organizational efforts from public and private sectors should be integrated and be reflected in government and regional policies on the multi-territorial scale so as to realize a symbiotic Earth where human society and Anthropocene ecosystems can both survive and evolve through collaborative interactions.

**Supplementary material 2**

**
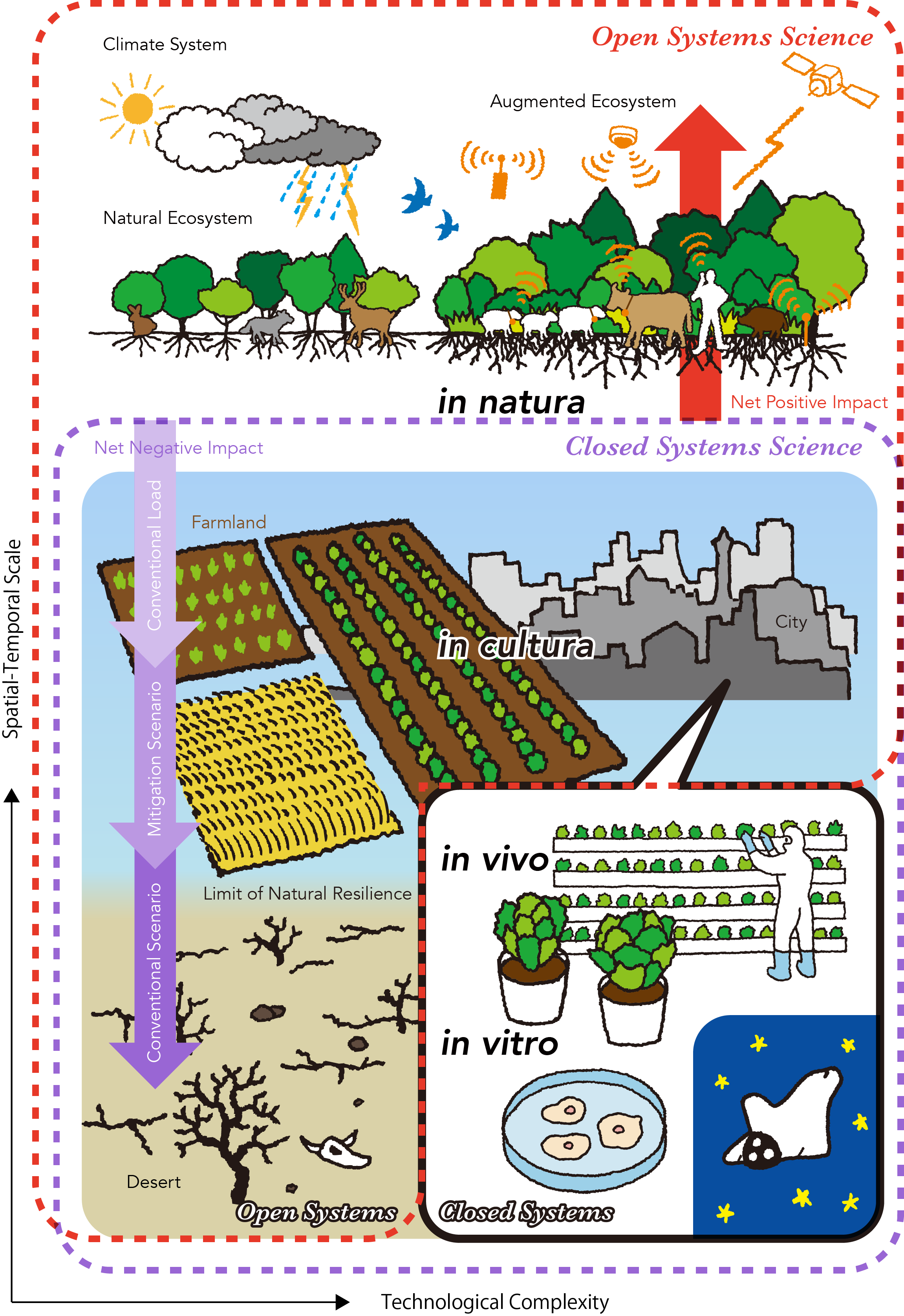
**

**Figure 1 legend (enlarged)**

Scale of scientific and industrial domains and associated human impacts on food production. Horizontal axis represents the degree of technological complexity required for realization. Vertical axis is the spatial-temporal scale involved for the maintenance from small (bottom) to large (top) scale, in which experimental systems in science and production modes in industry can be represented as *in vitro, in vivo, in cultura,* and *in natura* conditions.

Most biological studies are conducted with *in vitro* and *in vivo* experiments in confined environments, while public health studies such as cohort analysis focus on *in cultura* products without questioning the foundation of agriculture. Agronomy focuses on the optimization *in cultura*, such as high-precision resource management and genetically modified cropping systems, accelerating urbanization supported by the large-scale monoculture system, while ecology mainly treats preservation and conservation *in natura*. As a solution for future food production, anthropogenic augmentation of ecosystems is situated at the top right, which combines enhanced agricultural biodiversity with the support of information and communication technologies (ICT), making use of various biological resources in dense and mixed polyculture situations without external material inputs.

**Supplementary material 3**

**
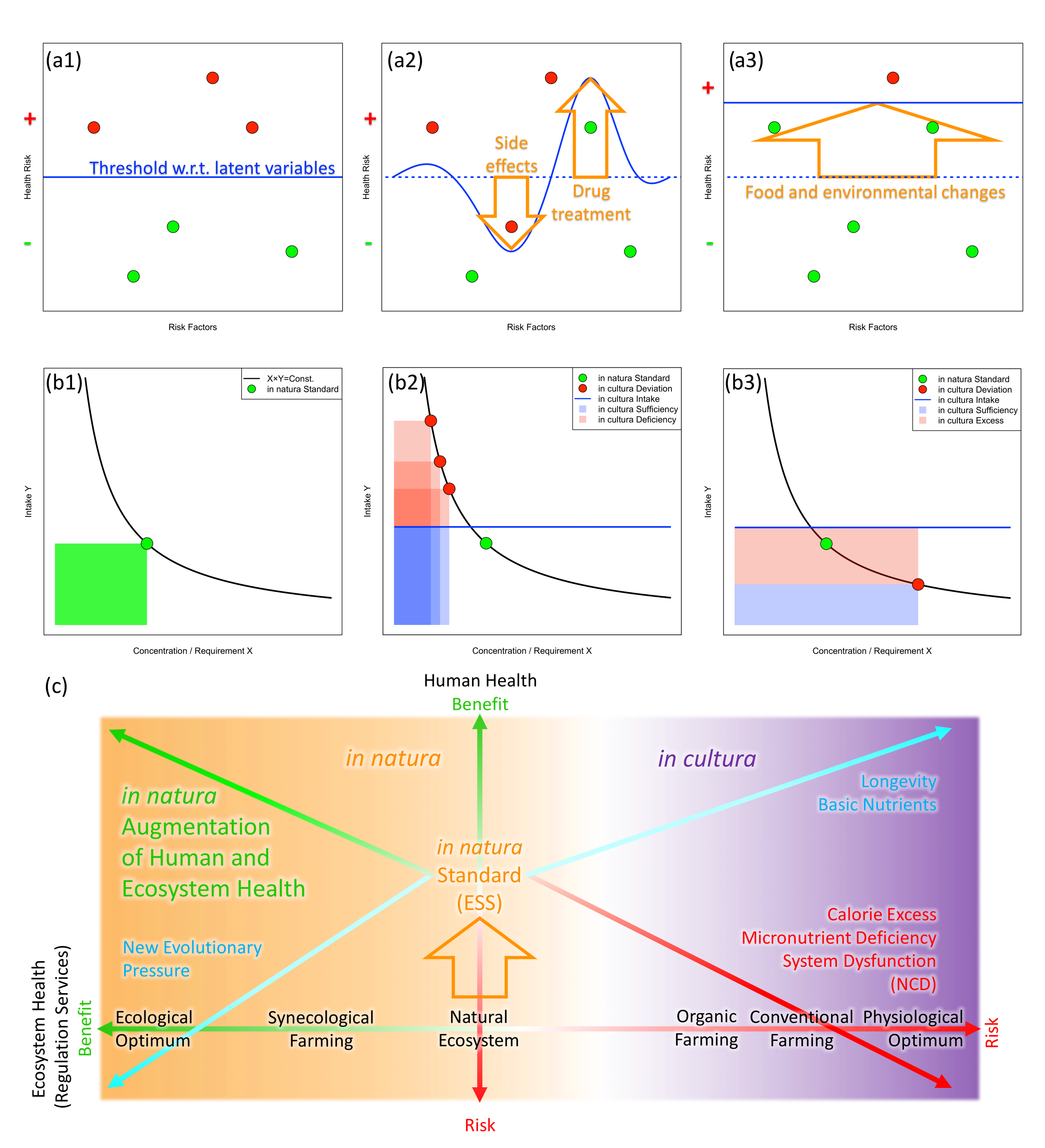
**

**Figure 2 legend (enlarged)**

(a1-a3): “Hidden reef model” that integrates observable (red and green circles) and latent (blue line) variables in biological study.

(a1): Observable elements of an organism (genotype, nutrition, biomarkers, etc.) are aligned horizontally with the value in vertical axis, normalized as risk (+, red circles) and beneficial (-, green circles) factors with respect to metabolic state conditioned by the net effect of latent variables (blue line).

(a2): Example of drug treatment and associated side effects.

(a3): Example of ideal treatment in relation to the change in environmental factors.

(b1-b3): Balance model of food variables with respect to evolutionary stable state (ESS, set as the green circles). X-axis is the concentration (content per unit weight) of food variable divided by physiological effects or environmental requirement for production. Y-axis is actual amount of the food intake.

(b1): The case of *in natura* ESS, set as the standard for (b2, b3).

(b2, b3): Example of conversion to *in cultura* environment: micronutrient deficiency (left shift of red circles in b2) associated with calorie overtake (right shift of a red circle in b3). The terms sufficiency, deficiency, and excess in the legends describe the signification of blue and red rectangle surfaces when X values represent the concentration per physiological requirement of the food variables. As a different application, if we discuss the environmental impact of food by plotting the value of concentration per environmental requirement on X, these surfaces should be reinterpreted as the amount of necessary (blue rectangles in b2 and b3), reduced (red rectangles in b2), and excess (red rectangle in b3) loads, respectively.

(c): Relationship between human and ecosystem health and farming methods.

Horizontal axis aligns the farming methods from the physiological (right) to ecological (left) optimum of plant communities, which corresponds to fewer (right) to higher (left) regulation services with high risk (red) and benefit (green) to ecosystem health.

Vertical axis is the state of human health or that of a single species, from risk (bottom, red) to benefit (top, green), which converges to ESS on the benefit side through evolution (orange arrow), conforming to (a3). Through the historical development of farming systems toward the physiological optimum of a single crop, human longevity and basic nutrients in the food matrix have been dramatically improved (upper right cyan arrow), while problems of micronutrient deficiency and calorie excess became newly dominant as system dysfunction, typically pervading non-communicable diseases (NCD) (lower right red arrow).

On the other hand, intensification toward the ecological optimum beyond the natural state, such as synecological farming (ref. 9), creates a new integrative approach that has the potential to address both human and ecological health as positively interacting solutions (upper left green arrow). The augmentation scenario may be associated with new evolutionary pressure on the human population reflected by demographic transition (ref. 99) and food and ecosystem changes (lower left cyan arrow). Conflicting directions between human and ecosystem health are indicated by cyan arrows.

**Supplementary material 4**

**
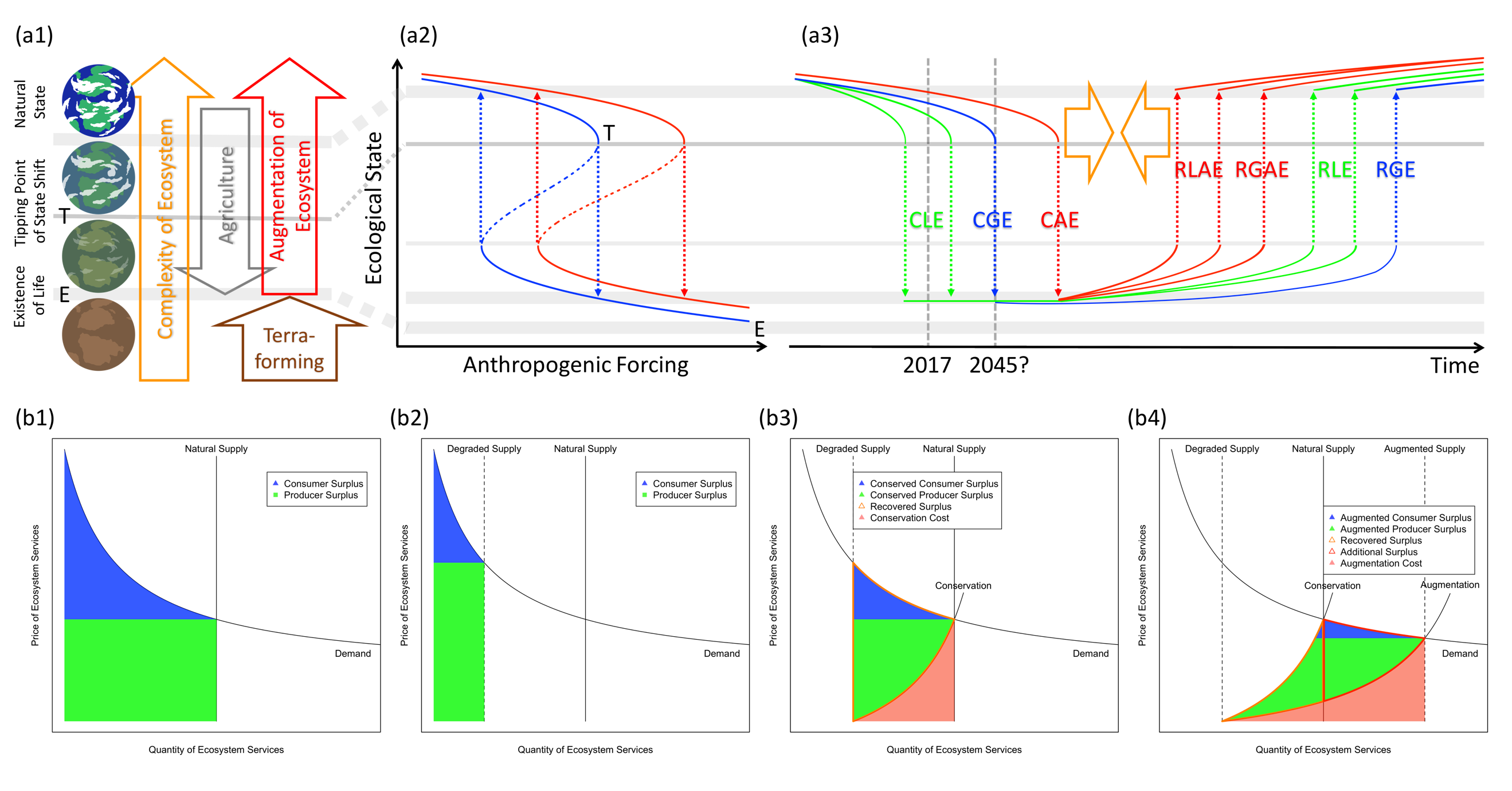
**

**Figure 3 legend (enlarged)**

Possible scenario of prevention and reversal of ecological state shift (a1-a3) and expected outcome on ecosystem services (ES) (b1-b4).

(a1): Relation between the evolution of Earth system, agricultural degradation, terraforming, and augmentation of the ecosystem with respect to ecological state.

(a2): Typical phase diagram of the ecological state shift with conventional (blue) and augmentation (red) scenarios in response to anthropogenic forcing such as agricultural land conversion, industrial pollution, and urbanization.

(a3): Estimated dynamics of the prevention and reversal of ecological state shift.

(b1): Pricing of ES in case of natural supply without human-induced degradation.

(b2): Pricing of ES in case of degraded supply under anthropogenic forcing.

(b3): Pricing of ES in a conservation scenario where humans pay a cost for the recovery of natural ecosystems to the level of natural supply.

(b4): Pricing of ES in the augmentation of ecosystems beyond conservation.

**Supplementary material 5**

**Examples that can be integrated in the hidden reef model in Fig. 2 (a1-a3).**

R: refers to the reef as observables, while S: represents the sea surface as latent variables.

R: A single-nucleotide polymorphisms study on the genetics of rheumatoid arthritis detected 98 biological candidate genes as risk factors, S: showing different variability between Asian and European ancestries^95^, while S: dietary profiles that could have provided a case-wise prevention effect^96^ were not included.

R: Gout culprit was often highlighted in food items with high content of purine, though S: endogenously synthesized purine and renal transport could be more important for the maintenance of uric acid pool in the body^97, 98^.

R: Australian Aborigines are known to be genetically susceptible to type 2 diabetes^99^, but S: the epidemiological evidence is also highly associated with the conversion to a Western diet and lifestyle^100^.

R: Identical twins inherit genetically identical risk factors, but even so, S: concordance on the occurrence of non-communicable diseases is not necessarily high: notably less than 5% for diabetes, rheumatoid arthritis, stroke, multiple sclerosis, and Crohn’s disease in dizygotic twins^101^.

R: Domestic animals *in cultura* develop various chronic diseases, though S: wild animals *in natura* are known to make use of various plant resources and natural environment for the prevention^102^.

R: Children who lived in East Germany in the 1990s exhibited lower incidence of asthma and atopy compared to those growing up in the more developed West Germany, despite the fact that S: East Germany was exposed to far higher levels of pollution but in different economic and environmental circumstances^103^.

R: Claimed research findings with large-scale trials may often be nothing but S: an accurate measure of the prevailing bias^45^.

**Supplementary material 6**

**Three steps of scientific reductionism for clarifying complex systems phenomena.**


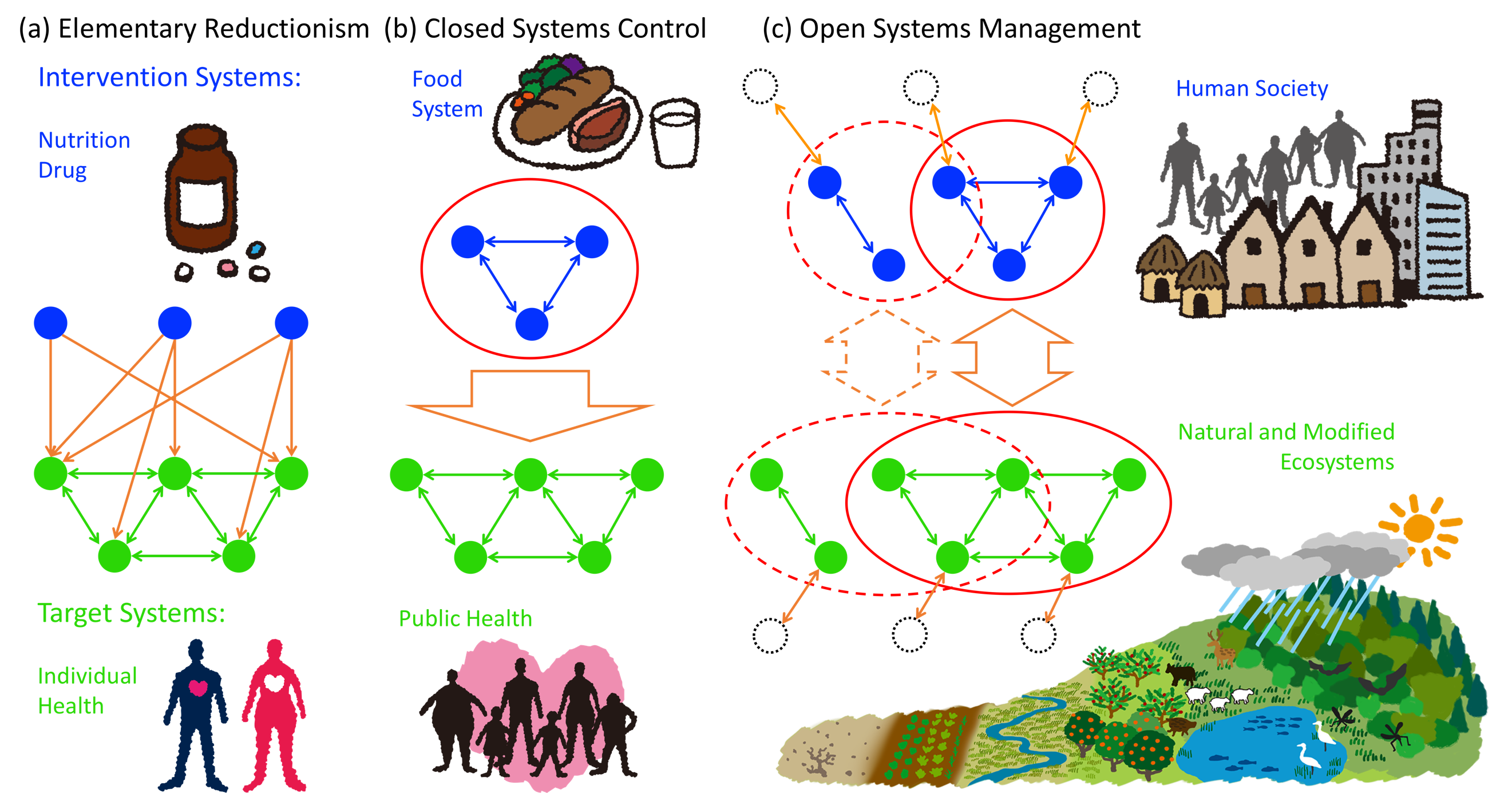


In the above figures, the target system subject to control is depicted with green elements with green arrows showing the interactions between them. The intervention system through which we control the target system is represented with blue elements and interactions, which also represent the modification of “sea surface” (blue line in Fig. 2 (a1-a3)). The effect of the intervention on the target system is shown with orange arrows.

Typical examples of target and intervention systems related to the food industry are included in each figure.

(a): Elementary reductionism where the target system is controlled with a single element. A typical example is the internal metabolic state of a human affected by nutrition supplements and drugs. Scientific methodologies are based on a single molecular determinant paradigm and it is possible to apply rigid statistical testing such as a randomized controlled trial (RCT). It can provide solutions to problems based on the deficiency and/or excess of the elements.

(b): Systems theory based on an isolated definition of the systems. Not only the target system but also the intervention form stable systems with constant internal dynamics. The consistency of dynamics enables modeling at the level of macroscopic system properties (as represented by red circles) such as mean values in the food matrix; traditional community-based food systems; standardized production methods of farming; systematized medical treatments; and other fixed protocol of intervention and subsequent examination of the comprehensive effect on the target system. Scientific methodologies call for the measurement of system-wide characteristics, such as the variation of system responses; hierarchical orders of correlation statistics^47^; and extraction of effective contexts where the system manifests desirable performances in response to a sequence of interventions. The macroscopic variables can include latent variables that escape from element-level analyses, as well as the emergent properties of system dynamics.

Newly approachable examples with this model include public health issues such as malnutrition and epidemic outbreaks in short time scale, and can provide partial solutions to such system-dysfunction problems.

(c): Management of open complex systems. Beyond scientific formalization, key real phenomena are mostly in the open environment and are dynamically changing their structure. The systems interact with other components in the outside environment (black dotted circles, which also correspond to latent variables (blue line) in Fig. 2 (a1-a3) and temporally change their composition (from dashed to solid red circles) through bidirectional interactions (orange arrows) between intervention and target systems. Rigorous modeling is limited in such situations, but it is still possible to refine the working hypothesis through constant renewal of the model with internal observation^12^. Measurements, statistical analysis, modeling, and human experience should work together in order to achieve better prediction and controllability^15^. The closed systems modeling in (b) is only an approximation of open systems to isolated subsystems with temporally stable structures.

Typical phenomena that can only be addressed with such open systems framework reside in long-term recursive interactions between human society and natural ecosystems, with highest complexity of system dysfunction risks: food system change including culture condition change, typically the transition between *in natura* and *in cultura* conditions^8^; lifestyle change in face of demographic change and industrialization^59^; food crises that emerge from the entanglement of various dynamic stresses, such as climate change and natural disasters, crop and market failure, poverty and insecurity, and political instability^104^; trans-generational and life course factors of chronic diseases^105^ and zoonoses^79^; and social-ecological effects of climate change ranging from recent global warming^32^ to human evolution^106^. Human augmentation of ecosystems (Fig. 1, top right) also becomes manageable with the open systems framework.

**Supplementary material 7**

**Examples of food variables and *in cultura* responses with respect to *in natura* ESS in Fig. 2 (b1-b3).**

Food variables that can be represented in Fig. 2(b1-b3) include major nutrients such as proteins, carbohydrates, lipids, vitamins, and minerals; micronutrients such as phytochemicals and trace elements; and non-nutrients such as dietary fiber, probiotics, and enzymes.

In addition to the trophic factors, thermodynamic parameters also form part of the variables: specifically, calorie content and the amount of entropy associated with the photosynthesis-driven water cycle in the Earth system that promotes the self-organization of the complex vegetation *in natura*^107^. Difference in the balance of energy and entropy flows in ecosystem cycles may be one of the distinctive factors between *in natura* and *in cultura* conditions that indirectly affect the secondary metabolite profiles of plants through the difference in ecological interactions^9^.

The variables also include any measurable quantity associated with food and its production: antibiotics, chemical residuals, fertilizer, plowing depth and frequency, plant size, growth stage and duration of crops, other meteorological variables involved (such as effects of climate change), surrounding biodiversity, nutritional effect of pollination^91^, soil microorganisms that affect gut microbiota, time length of evolutionary adaptation and modification of food habits^59, 106^, and other whole food effects.

In order to assess the interactions of the above variables, especially in changing environments, laboratory-based, element-wise experiments could further be expanded to explore the total responses in an open environment at the system level with a dynamical background^14^ (Supplementary material 6).

**Supplementary material 8**

**Examples of transition from conventional health-diet-environment trilemma to human augmentation of ecosystems scenario.**

Perhaps one of the best examples of the intersectionality of food production and laboratory evaluation is the current discourse over the global benefits of the vegetarian diet. Compared to the omnivore diet, a nutritionally reasonable vegetarian diet offers many opportunities to positively impact both human health and the global ecosystem^6, 24, 25^. In addition to reducing the use of hormones and antibiotics on dairy farms and cattle ranches, widespread adoption of a vegetarian diet could also reduce: (a) zoonotic infections by antibiotic-resistant microbes, (b) chronic disease associated with high-fat diets, (c) water pollution, (d) agricultural land usage (by 20–30%), and (e) greenhouse gases (by 30–60%).

Moreover, emerging international discourse on sustainable agroecosystems focuses on the benefits of increasing plant diversity to increase and stabilize yield and various ecosystem services^85^. This falls in line with the efforts to transform annual crops such as rice, wheat, and corn into perennial crops and to establish a low-input polyculture system. Perennial crops are more productive with less input and sequester more atmospheric CO2 than annuals. Turning cereal crops (all annuals) into perennials would have less of a negative impact on the environment and offer a wider accessibility to marginal environments^83, 84^.

Nevertheless, these perspectives are still under the constraint of the health-diet-environment trilemma. The benefits of the vegetarian diet are analyzed using databases of conventional agriculture based on tillage, fertilization, and possible use of chemicals (i.e., *in cultura*), which is still mostly focused on the trade-off between productivity and biodiversity. The health risk of meat consumption is also based on the conventional stockbreeding system (*in cultura*), and bushmeat consumption (i.e., *in natura*) is only criticized from the viewpoint of biodiversity protection^25^. The evolutionary nurtured diet system supports adaptation to foods of animal origin obtained in a natural environment (*in natura*)^106^. The vegetarian diet is a relatively new diet based on cultivated crops (*in cultura*). The agricultural products from large-scale monoculture production have just become increasingly dominant in less than a century. Even if short-term health benefits compared to *in cultura* meat products are reported with cohort studies over a dozen years in developed countries, contradicting evidence also exists, such as the health risks of plant-based carbohydrates and the benefit of animal-based saturated fat in terms of mortality and cardiovascular diseases in large cohorts including low and middle-income countries^108^. In order to reveal the long-term health effect, conformity to our genetic profile based on the evolutionary timescale should be thoroughly investigated. Adaptation to a cultured vegetarian diet for environmental reasons may be in conflict with the human genetic profile and evolutionary nurtured food habits, including trans-generational effects such as increased risks of adolescent substance misuse in offspring^63^.

The concept of an agroecosystem with increased plant diversity and perennial crop polyculture mimics the self-regulatory functioning of a natural ecosystem such as grassland (*in natura*), but further work is needed to implement a total design of agricultural production including crop choice strategy, management cost, and accessibility to smallholders who occupy the majority of world farms^29,35,36^.

The overall approaches lack the dimension of the augmentation of ecosystems and remain within a better conservation framework with combinations and rotation of only a few to a dozen species in cropping systems. Existing meta-analyses are based on the patchwork of diversity experiments in natural grassland and cropping systems, which contains various trade-offs among ecosystem services and costs to further integrate into a unified farming framework (e.g., between yield and pest suppression^109^ and between the extrapolation of grassland diversity effects and management costs of diversified harvests in cropping systems^84, 85^). Here, a naturally preserved ecosystem is the upper stream source of conception^83, 85^, and further intensification on the functioning of an ecosystem towards *in natura* higher organization is not considered. One crucial reason is that the tillage that perturbs the development of underground ecosystems is always present in farming design (i.e., principal factor of transition to *in cultura* environment), even in a reduced or less frequent form, at the foundation of agroecosystem studies aiming for sustainable production. However, maintaining the ecological optimum (*in natura*) generally nurtures topsoil and prevents erosion, which would protect an estimated 1/4 of global natural soil nitrogen sources and accelerate the positive cycle of atmospheric carbon and nitrogen fixation in a land ecosystem^110^. In the current sustainable agroecosystem approaches, the degree of an ecosystem’s self-organization and the biodiversity involved is still situated somewhere between a natural ecosystem and a monoculture condition, as typically represented by the multiple variants of organic farming in Fig. 2 (c). Compared to an augmentation strategy such as synecological farming, there is still an overall omission of long-term development of soil structure and ecological successions achievable in no-tillage conditions, which can further be extended by the introduction of species diversity beyond the level of natural ecosystems^9, 60, 61, 66^. In such a strict and enhanced ecological optimum condition (*in natura*), intensive diversification and various mixed associations of useful plant species become necessary to cope with diversity riot and to achieve a high yield level with portfolio strategies^65^. Newly developed perennial varieties and mutually beneficial combinations of crops should be extensively integrated in a wider spatiotemporal framework of biodiversity management through the *in natura* development of agroecosystems in such a way that is accessible to and livable for smallholders.

To achieve the augmentation scenario, strict substitution of tillage, fertilization, and chemical use with ecosystem functions is necessary to maintain and promote ecological optimum formation of an agroecosystem. The conventional methodologies can be totally replaced in small scale plots with positive disturbance, niche formation, and food chain construction^9, 61^. Especially in arid ecosystems, a strong biotic-abiotic feedback loop^18^ and local facilitation between plants^111^ are intrinsic to the structure of ecological regime shifts such as desertification. The input resources for abiotic amelioration are limited, and adverse effect may become greater. For example, destroying biotic interactions by ploughing could have fatal consequences on the restoration process^112^. Therefore, the augmentation of ecosystems in the global perspective must resolve the intensification of ecosystem cycles mainly through positive biotic intervention such as extensive introduction of genetic plant resources. Indeed, the diversity of plant species is the major factor that supports ecosystem multi-functionality in global drylands^17^. The augmentation of crop species diversity should be performed with several orders of magnitude more than the agrobiodiversity confined in a single mode of production such as intercropping and companion planting^66^.

Furthermore, in the augmentation scenario, it is still conceivable to invent a novel form of stockbreeding (different from conventional ones and uncontrolled bushmeat hunting) in a way that promotes ecosystem cycles and is compatible with the food habits of humans in the evolutionary timescale. A good starting point would be to combine the positive effects of pastoralism and management-intensive grazing in marginal land^86, 87^ with the *in natura* formation of a feeding environment and higher diversity of livestock and wild game.

Another factor that appears to distinguish the augmentation from conservation is the spatial pattern of vegetation patches. Ecological optimum formation with local facilitation is known to converge to the power-law distribution of patch patterns, in contrast to the regular and planned configuration of conventional agroecosystems^9, 65^. Maintaining the power-law distribution is also crucial to prevent desertification^19^, and the deviation to periodic patterns is considered an early warning signal^113^. A sustainable agroecosystem should also introduce and adapt the management to power-law patch distribution of crops, which is a visible proof of *in natura* self-organization.

Ecological modeling may play an important role in the prediction of regime shift^1, 18, 19, 111, 113, 114^. However, in order to guarantee the objectivity of research by eliminating *ad hoc* industrial factors, most of the work related to ecosystem multi-functionality is limited to protected and experimental areas, which are separated from actual developmental conflicts where various and extensive human interventions are inherent at the base line. Sound ecological modeling can provide warnings, but is not sufficient to prevent state shift caused by the environmental impact of agriculture because it does not provide accessible alternative solutions for primary food production, which makes it difficult to establish legal regulations strong enough to avoid the negative tipping point and secure food production.

Actually, in regions where ecosystems are at the brink of collapse, such as sub-Saharan Africa, the decrease of vegetation cover is substantially due to agricultural land conversion as opposed to the effect of climate change^115^. Despite the increasing contingency in weather events, long-term elevation of CO2 level is in fact estimated to recover vegetation cover in global dryland^116^, which contradicts the actual threats to livelihood such as deforestation by local developmental pressures and crop failure/food crisis, which are susceptible to negative effects related to increased population growth^104^.

The standard viewpoint of ecologists that “better prediction leads to better decisions” (e.g., ref. 114) is not by itself proactive and integrative enough to address present developmental conflicts because we essentially need concrete strategies and alternatives to convert primary food production into ways that reconstruct ecological diversity in order to increase resilience and sustain human health, which is out of the scope of ecological modeling. The true principal cause, namely, the fundamental trade-off between biodiversity and productivity in primary food production, needs to be resolved with the implementation of augmentation strategies down to the grass-root level, and by transforming the majority of environmental pressure produced by agriculture sectors into positive drivers of ecosystem reconstruction.

The prediction of the future ecological state and the transformation of food production into sustainable modalities should work in parallel, with greater and urgent importance placed on the substitution process of conventional agriculture. The complexity of social-ecological interactions in the augmentation scenario (i.e., the open systems in supplementary material 6 figure (c)) does not allow us to simply separately combine simulated projections of human activities and reactions of the climate system (i.e., the closed systems in supplementary material 6 figure (b)), although this is mainly what has been done in the prediction of global warming scenarios by IPCC^31^. Furthermore, the reactions of vegetation seasonality are much more complex than those related to the climate system, and we are also faced with the impossibility of estimating future trends^117^, which calls for the possibility of measurement with crowd-sourced solutions such as citizen science ^13, 14, 16^. Once successful augmentation of ecosystems takes place, predictions based on past data should be completely revised upward, and further prediction will require new investigation into emerging mechanisms that may be in a different alternative stable state of ecosystem.

The ecology of augmented ecosystems, which includes multi-scale interventions and interactions between human society, technological solutions, legal frameworks, natural and modified ecosystems, and the health profiles of humans and wildlife, must be established through real-time on-site management of open complex systems^12, 13^.

**References**

1. Okada, Y. *et al*. Genetics of rheumatoid arthritis contributes to biology and drug discovery. *Nature* **506,** 376-381 (2014).
2. Pattison, D. J., Harrison R. A. & Symmons, D. P. The role of diet in susceptibility to rheumatoid arthritis: a systematic review. *J. Rheumatol*. **31,** 1310-1319 (2004).
3. Richette, P. & Bardin, T. Purine-rich foods: an innocent bystander of gout attacks? *Ann. Rheum. Dis.* **71,** 1435-1436 (2012).
4. White, D., Lawson, N., Masters, P. & McLaughlin, D. *Clinical Chemistry*. (Garland Science, 2016).
5. Busfield, F. *et al*. A Genomewide Search for Type 2 Diabetes–Susceptibility Genes in Indigenous Australians. *Am. J. Hum. Genet.* **70,** 349–357 (2002).
6. O’Dea K. Diabetes in Australian Aborigines: impact of the western diet and life style. *J. Intern. Med.* **232,** 103-117 (1992).
7. Wong, A. H., Gottesman, I. I. & Petronis, A. Phenotypic differences in genetically identical organisms: the epigenetic perspective. *Hum. Mol. Genet.* **14,** R11-R18 (2005).
8. Engel, C. *Wild Health: Lessons in Natural Wellness from the Animal Kingdom.* (Mariner Books; Reprint edition, 2003).
9. Graham-Rowe, D. Lifestyle: When allergies go west. *Nature* **479,** S2–S4 (2011).
10. FAO (Food and Agriculture Organization). Global report on food crises 2017. http://www.fao.org/3/a-br323e.pdf (FAO, 2017).
11. Kuh, D. L. & Ben-Shlomo, Y. *A Life Course Approach to Chronic Disease Epidemiology; Tracing the Origins of Ill-health from Early to Adult Life*. (Oxford University Press, 1997).
12. Verginelli, F., Aru, F., Battista, P. & Mariani-Costantini, R. Nutrigenetics in the light of human evolution. *J. Nutrigenet Nutrigenomics* **2**, 91-102 (2009).
13. Katsuki, A. in *Bioastronomy - The Next Steps Proceedings of the 99th Colloquium of the International Astronomical Union held in Balaton, Hungary, June 22–27, 1987* (eds, Marx. G.), Water: An Absolute requirement for Life. (Springer, 1988).
14. Dehghan, A. et al. Associations of fats and carbohydrate intake with cardiovascular disease and mortality in 18 countries from five continents (PURE): a prospective cohort study. *Lancet* **390**, 2050–2062 (2017).
15. Letourneau, D.K. et al. Does plant diversity benefit agroecosystems? A synthetic review. *Ecological Applications* **21**, 1, 9–21 (2011).
16. Houlton,B.Z., Morford,S.L. & Dahlgren, R. A. Convergent evidence for widespread rock nitrogen sources in Earth’s surface environment. *Science* **360**, 58–62 (2018).
17. Xu, C. et al. Local facilitation may cause tipping points on a landscape level preceded by early-warning indicators. *The American Naturalist* **186**, 4, E81-90 (2015).
18. Pueyo et al. Comparing Direct Abiotic Amelioration and Facilitation as Tools for Restoration of Semiarid Grasslands. *Restoration Ecology* **17**, 6, 908-916 (2009).
19. Kéfi, S. et al. Early Warning Signals of Ecological Transitions: Methods for Spatial Patterns. *PLoS One* **9**, 3, e92097 (2014).
20. Maris, V. et al. Prediction in ecology: promises, obstacles and clarifications. *OIKOS* **127**, 2, 171-183 (2018).
21. Gaiser, T. et al. Future productivity of fallow systems in Sub-Saharan Africa: Is the effect of demographic pressure and fallow reduction more significant than climate change? *Agric. For. Meteorol* **151**, 1120–1130 (2011).
22. Lu,X., Wang, L. & McCabe, M.F. Elevated CO2 as a driver of global drylands greening. *Sci. Rep.* **6**, 20716, doi: 10.1038/srep20716 (2016).
23. Xu, L. et al. Temperature and vegetation seasonality diminishment over northern lands. *Nat. Clim. Change.* **3**, 581–586 (2013).
